# The molecular epidemiology of a dengue virus outbreak in Taiwan: population wide versus infrapopulation mutation analysis

**DOI:** 10.1371/journal.pntd.0012268

**Published:** 2024-06-13

**Authors:** You-Yuan Tsai, Dayna Cheng, Sheng-Wen Huang, Su-Jhen Hung, Ya-Fang Wang, Yih-Jyh Lin, Huey-Pin Tsai, Justin Jang Hann Chu, Jen-Ren Wang

**Affiliations:** 1 Department of Medical Laboratory Science and Biotechnology, College of Medicine, National Cheng Kung University, Tainan, Taiwan; 2 Department of Pathology, National Cheng Kung University Hospital, Tainan, Taiwan; 3 Institute of Basic Medical Sciences, College of Medicine, National Cheng Kung University, Tainan, Taiwan; 4 National Mosquito-Borne Diseases Control Research Center, National Health Research Institutes, Tainan, Taiwan; 5 Division of General Surgery, Department of Surgery, College of Medicine, National Cheng Kung University Hospital, Tainan, Taiwan; 6 Infectious Diseases Translational Research Program and Department of Microbiology and Immunology, Yong Loo Lin School of Medicine, National University of Singapore, Singapore, Singapore; 7 Center of Infectious Disease and Signaling Research, National Cheng Kung University, Tainan, Taiwan; Solena Ag, UNITED STATES

## Abstract

Dengue virus (DENV) causes approximately 390 million dengue infections worldwide every year. There were 22,777 reported DENV infections in Tainan, Taiwan in 2015. In this study, we sequenced the C-prM-E genes from 45 DENV 2015 strains, and phylogenetic analysis based on C-prM-E genes revealed that all strains were classified as DENV serotype 2 Cosmopolitan genotype. Sequence analysis comparing different DENV-2 genotypes and Cosmopolitan DENV-2 sequences prior to 2015 showed a clade replacement event in the DENV-2 Cosmopolitan genotype. Additionally, a major substitution C-A314G (K73R) was found in the capsid region which may have contributed to the clade replacement event. Reverse genetics virus rgC-A314G (K73R) showed slower replication in BHK-21 and C6/36 cells compared to wildtype virus, as well as a decrease in NS1 production in BHK-21-infected cells. After a series of passaging, the C-A314G (K73R) mutation reverted to wildtype and was thus considered to be unstable. Next generation sequencing (NGS) of three sera collected from a single DENV2-infected patient at 1-, 2-, and 5-days post-admission was employed to examine the genetic diversity over-time and mutations that may work in conjunction with C-A314G (K73R). Results showed that the number of haplotypes decreased with time in the DENV-infected patient. On the fifth day after admission, two new haplotypes emerged, and a single non-synonymous NS4A-L115I mutation was identified. Therefore, we have identified a persistent mutation C-A314G (K73R) in all of the DENV-2 isolates, and during the course of an infection, a single new non-synonymous mutation in the NS4A region appears in the virus population within a single host. The C-A314G (K73R) thus may have played a role in the DENV-2 2015 outbreak while the NS4A-L115I may be advantageous during DENV infection within the host.

## Author summary

Dengue virus (DENV) is an RNA virus which causes many infections worldwide. Taiwan experienced the largest DENV-2 outbreak in 2015 which resulted in many fatalities. Specific reasons for the increase in severity during this outbreak remains unclear. The molecular epidemiology of this outbreak showed that clade replacement may have occurred, with the 2015 isolates forming a new clade and the old clade consisted of sequences from 2001–2004 in Taiwan. A dominant C-A314G (K73R) substitution was found in the 2015 DENV-2 outbreak isolates, which may have resulted in a clade replacement in the DENV-2 Cosmopolitan genotype. Further analysis of genetic variation of the virus population of one DENV-2 infected patient showed a decrease of haplotypes during the progression of infection and an NS4A-L115I mutation that did not appear until a later time-point. These show the difference between the variations found in a population wide analysis compared to an infrapopulation analysis which can influence viral infection.

## Introduction

Dengue virus (DENV) belongs to the genus *Flavivirus* in the *Flaviviridae* family and it is a positive-sense single stranded RNA (+ssRNA) virus consisting of approximately 10,700 nucleotides and encodes a 3,411 amino acid long precursor polyprotein [1]. The viral genome encodes three structural proteins (capsid (C), premembrane (prM), envelope (E)) and seven non-structural proteins (NS1, NS2A, NS2B, NS3, NS4A, NS4B, NS5). DENV consists of four serotypes (DENV-1 to 4) [2] and all four DENV serotypes have emerged from sylvatic strains in the forests of Southeast Asia [3]. Clinical manifestations of DENV viral infections range from asymptomatic to severe illness that may lead to death if not carefully managed. The symptomatic cases are categorized as undifferentiated febrile illness (UF), dengue fever (DF), dengue hemorrhagic fever (DHF), dengue shock syndrome (DSS) and unusual dengue (UD) or expanded dengue syndrome (EDS) [4].

DENV, like other RNA viruses, tend to have a high mutation rate due to the low fidelity RNA dependent RNA polymerase (RdRp) which lacks proofreading activity, resulting in an estimated error rate during replication of 10^−3^ to 10^−5^ mutations per nucleotide per replication cycle [5,6]. For infections with RNA viruses, an acutely infected organism can harbor 10^9^ to 10^12^ viral populations at any time [5]. With an RNA virus genome length of approximately 10^4^ nucleotides, every possible single mutation or many double mutations are likely to occur. Most mutant viruses are probably defective, but the produced virus population potentially contains diverse viruses. Viral populations that are genetically linked through mutations generated during replication and interact in a cooperative manner to contribute to the overall characteristics of the population define the concept of quasispecies [7,8]. RNA viruses cause both acute and chronic infections. Acute infection viruses tend to have a lower chance of producing mutant viruses. While it is understandable that viral quasispecies plays an important role in chronic infectious disease because the course of these diseases is very long [9–14], recent reports suggest the importance of genetic diversity of viral populations in acute viral infectious diseases [15–17]. Wang et al. previously reported that DENV was found to be presented as a population of closely related genomes *in vivo* in plasma [18]. In a study done by Vignuzzi *et al*., quasispecies was found to be essential for acclimating to and enduring new selective pressures in varying environments due to the increase in genetic diversity of the viral population [15]. The cooperation amongst the viral variants allows for successful colonization of the new ecosystem. Their data provided evidence for a fundamental prediction of the quasispecies theory and established a link between population dynamics, mutation rate, and pathogenesis. There are accumulating reports demonstrating the importance of quasispecies of RNA viruses in the progression of disease. Using next generation sequencing (NGS) analysis, previous study on DENV-2 in the 2015 outbreak in Taiwan identified variations in the viral population in the non-structural protein region that showed better growth kinetics, thermal stability and sensitivity in comparison to wildtype viruses, thus may have played a role during the outbreak [19]. In a single enterovirus A71 (EV-A71) autopsy patient, due to a shift in the major haplotype, a VP1-31 D-to-G mutation was found in the viral population that can overcome the selective pressure, allowing the virus to successfully infiltrate the central nervous system [20].

DENV has caused many outbreaks throughout Southeast Asia since the 1950s [21–23]. Most recently within the last two decades, DENV epidemics have occurred in countries such as Singapore [24–26], China [27–31], Vietnam [32,33], Thailand [34], and Taiwan [3,35–37] to name a few. In the history of Taiwan, several dengue epidemics had been documented in the first half of the twentieth century and the most severe one occurred in 1942–43 when an estimated five-sixths of the population on the island was infected [3]. Since then, no dengue outbreaks had been reported in Taiwan until 1987. Dengue epidemics with various sizes have been found in southern Taiwan almost every year, mainly in Kaohsiung City [3,36,38]. However, while DENV is prevalent in Taiwan, it is currently not considered to be endemic in Taiwan [36]. Taiwan has experienced many outbreaks; however, Taiwan was faced with two consecutive DENV outbreaks in 2014 and 2015. The 2015 DENV-2 outbreak was by far the greatest and most severe dengue outbreak which Taiwan had ever encountered. This outbreak resulted in over 43,000 dengue cases, including 228 deaths. with 22,777 reported DENV infections in Tainan, Taiwan [35,37,39].

Molecular surveillance of DENV is important in identifying potential mutations that may be associated with outbreaks. Additionally, apart from a population wide analysis, understanding the virus genetic diversity during the course of an infection is also vital, and may help to identify mutations that may drive the progression of disease severity in DENV infection since this remains unknown. In this study, the molecular epidemiology of DENV and the genetic variation of capsid (C), pre-membrane (prM), and envelope (E) genes of DENV isolates were analyzed. In addition, based on NGS data, the analysis of the DENV haplotypes and the genetic variations found in RNA sequences during DENV replication in human were explored.

## Materials and methods

### Ethics statement

This study was conducted in Tainan, Taiwan. The waiver was approved by Institutional Review Board (IRB) of National Cheng Kung University Hospital (No. B-ER-104-228). The clinical data were anonymized and de-identified prior to analysis. Thus, informed consent was not obtained from patients prior to the study.

### Patient samples

A total of 1,581 patients’ serum samples with suspected DENV infections were collected at National Cheng Kung University Hospital (NCKUH) in 2015. DENV-2 (146), DENV-1 (1), and DENV-3 (2) were successfully isolated by using *Aedes albopictus* C6/36 cells. For a population wide analysis, DENV-2 isolates were randomly selected based on disease severity, viral load, and symptoms (S1 Table). A total of 45 DENV-2 isolates were thus randomly selected for sequencing and phylogenetic analysis, of which 42 isolates were classified as primary infections and 3 isolates were from secondary infections (S2 Table). For an infrapopulation analysis, three clinical samples obtained from one patient infected with DENV-2 in 2016, one sample from the only DENV-1-infected patient in 2015, and two samples from the only DENV-3-infected patients in 2016 and 2017 were analyzed via next-generation sequencing (NGS).

### Cells and virus isolations

*Aedes albopictus* C6/36 cells (ATCC: CRL-1660) were cultured in RPMI with 10% fetal bovine serum (FBS) and 2% penicillin/streptomycin (P/S) at 28°C. Both baby hamster kidney cells (BHK-21, ATCC: CRL-12071) and Vero cells (ATCC: CCL-81) were cultured in Eagle’s Minimum Essential Medium (EMEM) with 10% FBS, 2% P/S, and 1% sodium pyruvate at 37°C. C6-36 cells were used in virus isolation, BHK-21 cells were used in transfection, virus culture, virus stability assay, and virus growth kinetics, and Vero cells were used in ELISpot assay to obtain virus titers.

The real-time PCR positive samples were used for virus isolation. C6/36 cells were incubated with 100 μl serum specimen in 500 μl RPMI viral medium (2% FBS, 2% P/S) at 28°C for at least 1 hour, washed twice with PBS, followed by the addition of 1.5 mL RPMI viral medium. The cultures were incubated for 3 days and were examined daily for cytopathic effect (CPE) such as cell swelling, fusion, and vacuolization. The production of dengue viruses was determined using indirect immunofluorescence assay (IFA) by anti-DENV Ab GTX29202 (GeneTex). The IFA negative specimen was further passaged in C6/36 cells for 3 days, and successful DENV production was determined by IFA. The supernatants of the IFA positive samples were harvested and stored at -80°C.

### Virus extraction, C-prM-E gene amplification, and nucleotide sequencing

Viral RNA was extracted from C6/36 cells infected with DENV isolates using QIAamp Viral RNA Mini kit (QIAGen, Cat. No. 52904). Primers designed to amplify and sequence C, pre-membrane (prM), and E gene sequences are shown in S3 Table. Two sets of primers, D2-14F/D2-1572R and D2-1157F/D2-2610R, were used for RT-PCR to amplify DENV-2 sequences using QIAGEN OneStep RT-PCR Kit (QIAGen, Cat. No. 210212). The OneStep RT-PCR reactions were performed at 50°C for 30 mins, 95°C for 5 mins, 35 cycles of 95°C for 30 seconds, 50°C for 30 seconds, and 72°C for 2 minutes, and a final extension step of 72°C for 10 mins. The PCR products were purified using the Qiagen QIAquick Gel Extraction kit (QIAGen, Cat. No. 28706). Nucleotide sequences were determined with the ABI Prism automated DNA sequencing kit and the ABI Prism 3700 DNA sequencer (Applied Biosystems, Foster City, CA, USA) according to the manufacturer’s protocols. Overlapping nucleotide sequences were combined for analysis and edited with the Invitrogen Vector NTI Advance Sequence Analysis Software package (Thermo Fisher Scientific Inc.). Sequences were uploaded to GenBank under the accession numbers OR618341-OR618363 and OR593362-OR593383.

### Bayesian evolutionary analyses

To estimate the nucleotide substitution rates and the time to the most recent common ancestor (TMRCA), a total of 45 DENV-2 C-prM-E gene sequences were used in this study for phylogenetic analyses. Of these 45 sequences, 22 sequences were obtained from previous DENV-2 next-generation sequencing (NGS) selected from 2015 cases (GenBank: OR593362-OR593383) [19], and the remaining 23 sequences were newly sequenced in this project, also selected from the 2015 outbreak cases (GenBank: OR618341-OR618363). All 45 DENV-2 sample sequences were isolates of indigenous cases. This analysis also included 20 DENV-2 sequences of Taiwan isolates selected from representative strains of imported cases and indigenous cases from major local DENV-2 outbreaks from 1944–2015, combined with 18 DENV-2 global reference sequences (Accession No. EF105384, AY702040, AF100469, AB122022, HQ026763, M29095, KM587709, GU131924, NC_001474, U87411, DQ44823, KP757112, AY037116, JF327392, KT187555, MF004385, KU509270, and MK564477) of different genotypes available in GenBank. Nucleotide sequences were aligned using Clustal W software [40]. The datasets were analyzed using the Bayesian Markov Chain Monte Carlo (MCMC) method in the BEAST 2 package [41].

The best-fit substitution models were determined on the ATGC bioinformatics platform using the Phylogenetic Maximum Likelihood (PhyML) utility [42]. Automatic model selection was performed by Smart Model Selection (SMS) based on the Akaike Information Criterion (AIC) and Bayesian Information Criterion (BIC) within PhyML [43]. The dataset was evaluated using a relaxed uncorrelated lognormal molecular clock (local clock model) and a strict clock (global clock) with a Bayesian skyline coalescent prior [44]. Procedures were run for 60,000,000 generations, and the parameter values were sampled after each 6,000 steps. The final log and tree files were combined using Log- Combiner v2.7.6, set with a 10% burn-in from each run. The combined results of the log files with an effective sample size (ESS) greater than 200 were analyzed and viewed using Tracer version 1.7.2. The combined trees were annotated by Tree Annotator v.2.7.6 and visualized in the FigTree 1.3.1 program.

### DENV full-length genome amplification and next-generation sequencing

Viral RNA was extracted from DENV-1, -2, and -3 isolates using the QIAamp Viral RNA Mini Kit (QIAGen) according to the manufacturer’s instructions. RNAs were reverse transcribed into cDNA using the SuperScript III Reverse Transcriptase (Invitrogen) or Maxima H Minus Reverse Transcriptase (ThermoScientific) with anti-sense primer Rv3 and Rv5 (S4 Table) [45]. Reverse transcription was performed at 50°C for 60 mins and 72°C for 15 mins, or at 50°C for 30 mins and 85°C for 15 mins by SuperScript III and Maxima H Minus, respectively. Two DNA fragments were prepared that covered the whole dengue genome using the KOD Plus Kit (TOYOBO). Primers Fw1 and Rv3 were used to amplify a template from the first fragment of the dengue genome (S4 Table). The primers Fw4 and Rv5 were used to amplify a template from the second fragment of the dengue genome. The PCR reactions for both fragments were performed at 95°C for 5 mins, followed by 30 cycles at 95°C for 1 min, 55°C for 1 min and 68°C for 8 mins (6 mins for the second fragment), and a final step at 68°C for 7 mins. Two fragments of PCR amplicon were purified by phenol/chloroform. More than 1 μg of DNA was sequenced by the Illumina Miseq platform for next-generation sequencing (NGS).

### DENV haplotypes analysis

NGS can sequence millions of small fragments of DNA in parallel and provide high depth to deliver accurate data and insight into unforeseen variations [46,47]. In recent years, NGS has been commonly used as a powerful tool for analyzing DENV genetic diversity in quasispecies. The full-length DENV consensus sequence was first assembled from the read datasets obtained from the NGS Illumina MiSeq platform, using Contig Express (Vector NTI software, v8) and reference sequences (DENV-1 GenBank: FJ176779, DENV-2 GenBank: KU365902, and DENV-3 GenBank: KF955461). The short NGS reads first underwent *de novo* assembly and were then aligned with the consensus sequence using RayAssembly software with the -k parameter set to 31. Subsequently, after alignment with the consensus sequence, predicted haplotype sequences were generated using a QuasiRecomb software (www.cbg.ethz.ch/software/quasirecomb), which able to infer quasispecies from deep-coverage NGS data as well as provide variation analysis data of each position [48]. The percentage of each predicted haplotype within the viral population of each sample was provided. Additionally, with QuasiRecomb, the data reads were refined to remove duplicate reads, leaving only the unique read sequences. Haplotype read sequences with a percentage of ≥1% were then further analyzed. Statistically significant differences of haplotype proportions and genetic variations were analyzed by MEGA 7 software. The full-length deep sequencing reads of DENV-1, DENV-2 and DENV-3 were deposited in the NCBI Sequence Read Archive under the BioProject ID PRJNA1020645, SRA Accession Nos. SRR26188978-SRR26188983, BioSample Accession Nos. SAMN37555429-SAMN37555434, and GenBank accession numbers OR618318-OR618323.

### Construction of infectious cDNA Clones

Site-directed mutagenesis PCR was performed to construct cDNA clones containing the substitution to be investigated. Using pDL-DENV2-EGFP-C60-10062016-A2 (pDENV2-1006) as template (originally pDV2-16681) [49], amplicons were amplified by PCR using primer pairs designed for site-directed mutagenesis (S5 Table). PCR reactions were 95°C for 5 mins, followed by the annealing phase consisting of 30 cycles of 95°C for 1 min, 65°C for 1 min, and 68°C for 5 mins, and the final extension phase at 68°C for 10 mins.

The amplicons were purified by gel extraction according to manufacturer’s protocol (Geneaid Cat. No. DF300). The amplified fragments were then overlapped, extended, and amplified by primer pairs SING-D2-Sac-I-F and SING-D2-ScaI-R. Overlapped mutant fragments were cloned into pGEM-T Easy Vector using T4 DNA Ligase (PROMEGA) and transformation was done using Xl1-blue competent cell on a Luria-Bertani (LB) agar plate containing 100 μg/mL ampicillin. The pDENV2-1006 plasmid and mutant plasmid were digested with restriction enzymes Scal-HF and SacI and purified by gel extraction. The digested mutant fragment and pDENV2-1006 plasmid were ligated using T4 DNA ligase and incubated at 16°C overnight. Ligated products were then transformed into One Shot Stbl-3 competent cells, spread on LB agar plates containing 35 μg/mL kanamycin and incubated in 29°C for 3 days. Colonies containing the plasmids were selected and cultured in 3 mL LB culture (35 μg/mL kanamycin) for 36–48 hours, then amplified further in 100 mL LB culture (35 μg/mL kanamycin) for another 36–48 hours. The plasmids were harvested using HiYield & Plasmid Mini Kit (RBC BioScience) for cultures of 3 mL LB and ZymoPURE II Plasmid Midiprep Kit (ZYMO) for cultures of 100 mL LB.

### Production of reverse genetics viruses

Mutant plasmid was transfected into BHK-21 cells (4 x l0^5^/well) in 6-well plates using PolyJet *In Vitro* DNA Transfection Reagent (SignaGen). Three μg of plasmid (2 μg C-A314G (K73R), 1 μg pTet-Off) was transfected into each well. After a 24-hour incubation period at 37°C, the medium of each well was refreshed with 2 mL EMEM (10% FBS, 2% P/S, 1% sodium pyruvate). Reverse genetics viruses were harvested 6–7 days post-transfection, or when cytopathic effect (CPE) and/or fluorescence were observed on more than 75% of cells. The viruses were then subcultured in BHK-21 cells (passage 1, P1 virus). P1 viruses were quantified by ELISpot for further analysis.

### Virus growth kinetics

In order to analyze the growth kinetics of the reverse genetics viruses, BHK-21 and C6/36 cells (2 x 10^5^/mL) were seeded in 24-well plates. After a 24-hour incubation period, the cells were infected with 100 μl of virus with MOI of 0.01 in virus culture medium (EMEM: 2% FBS, 2% P/S, 1% sodium pyruvate). Viruses were harvested at 0-, 1-, 2-, 3-, and 4-days post-infection. Collected virus samples were titrated by ELISpot to obtain viral titer.

### DENV NS1 ELISA Assay

The NS1 titer of DENV was quantitated by DENV NS1 capture enzyme-linked immunosorbent assay (ELISA). The anti-DENV NS1 monoclonal antibodies (mAbs), acting as capturing and detecting reagents for the different serotypes, were used in a sandwich ELISA procedure. Corning 96-well Clear Polystyrene High Bind Stripwell Microplates were coated with 5 μg/ml capturing DENV NS1 1D33 mAb (100 μl per well) in PBS and incubated overnight at 4°C. On the following day, plates were incubated with blocking buffer (1% BSA) for 1 hour at 37°C. The plates were then incubated with 100 μl of the supernatant of DENV-infected cells, positive control, negative control, or calibrator, and 100 μl of 2X LG-1 buffers for 1 hour at 37°C. Plates were washed three times with washing buffer (0.05% Tween 20 in dH_2_O). Detection antibody (100 μl DENV NS1 1D33+ 2H5-HRP (1:200) with 1X LG1) was added into each well and incubated for 1 hour at 37°C. The wash step was then repeated four times. After wash, 100 μl of TMB substrate was added into each well for colorimetric reaction and incubated for 5 minutes at 37°C. The reaction was stopped by adding 50 μl of 2N H_2_SO_4_. The plate was read at 450 nm absorbance by ELISA reader [50].

### ELISpot Assay

Enzyme-Linked ImmunoSpot assay (ELISpot) is designed for the quantitation of single secreting cell detection among 1,000,000 cells in a 96-well plate. Vero cells (2 x l0^4^/100μl/well) were seeded in a 96-well plate and cultured at 37°C, 5% CO_2_ for 24 hours. Cells were infected with 30 μl of ½ log serially diluted viruses. Virus adsorption was performed for 1 hour at 37°C and 5% CO_2_, after which 150 μl of overlay medium (EMEM virus medium, 1% methyl cellulose) was added into each well. After four days, the supernatant was aspirated, wells were washed twice with 180 μl of PBS, and cells were fixed with 100 μl of 80% methanol for 10 mins at room temperature. Once fixed, immunostaining was performed whereby the methanol was removed and 180 μl of antibody dilution buffer (5% milk in 1X PBS) was added to each well for blocking and incubated at room temperature for 30 mins. Cells were then incubated with 100 μl of MAB8705, mouse anti-dengue complex monoclonal antibody (Millipore, Burlington, MA, USA) at a 1:2000 dilution in antibody dilution buffer and incubated at 37°C for 2 hrs on an orbital shaker. The wells were washed 4 times with PBS-T (0.05% Tween 20 in PBS), incubated with 100 μl KPL-Goat anti-mouse IgG conjugate HRP (KPL #5220–0460, LGC Seracare, Milford, MA, USA) at 1:3000 dilution in antibody dilution buffer, and then incubated at 37°C for 1 hr on an orbital shaker. The wells were then washed 6 times with PBS-T and allowed to dry for a few minutes. Once dried, 60 μl of TrueBlue substrate (TrueBlue Peroxidase Substrate, KPL #5510–0030, LGC Seracare, Milford, MA, USA) was added to each well and incubated at room temperature for 20 mins in the dark. The number of spots and photographs of each well was then taken using the Immunospot system and software ImmunoSpot Image Acquisition and cell counting software (CTL-ImmunoSpot, Cleveland, OH, USA).

### Competition Assay

BHK-21 cells (1.5 x 10^5^/mL) were seeded in 24-well plates and incubated for 24 hours. Cells were infected with 1 x 10^5^ FFU of a 1:1 mixture of mutant virus and wildtype (WT) virus, mutant virus only, and WT virus only. Viruses were collected after more than 75% CPE or fluorescence was observed and repassed. Viral RNA was extracted from viral supernatant using QIAamp Viral RNA Mini Kit. Viral RNA was reverse transcribed into cDNA and amplified the fragments using QIAGEN OneStep RT-PCR Kit with the primer pairs SING-D2-3648-F (5’- GTT TCC TAA CAA TCC CAC CAA CAG C -3’) and SING-D2-3757-R (5’- ATC CTT CCA ATC TCT TTC CTG AAC C -3’). The PCR products were pooled and the fragments corresponding to the location of the variant were purified by PureLink Quick Gel Extraction and PCR Purification Combo Kit (Cat: K220010) and sequenced by Sanger sequencing (Genomics). Sequencing was done at passage 1 (P1), P5 and P10.

### Statistical analysis

Statistical difference of viral growth kinetics between mutant and wildtype virus was analyzed using two-way analysis of variance (ANOVA). A p-value of <0.05 was considered statistically significant. All data were analyzed by GraphPad Prism 5.

## Results

### Phylogenetic analysis of the DENV outbreak in 2015 in Tainan, Taiwan

To determine whether the sequence variation of DENV induced different clinical symptoms response in the patients, a total of 146 DENV-2 clinical isolates were successfully obtained from patients’ serum samples at NCKUH. Of the DENV-2 clinical isolates collected. From these isolates, a total of 18 mild cases, 10 severe cases, and 17 fatal cases were randomly selected for phylogenetic analysis, with samples being selected at random based on symptoms and viral load. Of the 45 total isolates selected for further analysis, 3 isolates were classified as secondary infections and were all fatal case isolates (S2 Table). Depending on the viral load, the 45 isolates were divided into high and low viral load groups (S1 Table). It was noted that while mild cases had a higher number of specimens in the low viral load group, it was the opposite for severe and fatal cases (S1A Fig). However, the percentages in the number of specimens in each group was similar for severe and fatal cases (S1B Fig). Additionally, in all three mild, severe, and fatal cases, there was a significant difference between the low and high viral load groups (S1C Fig).

**Fig 1:**
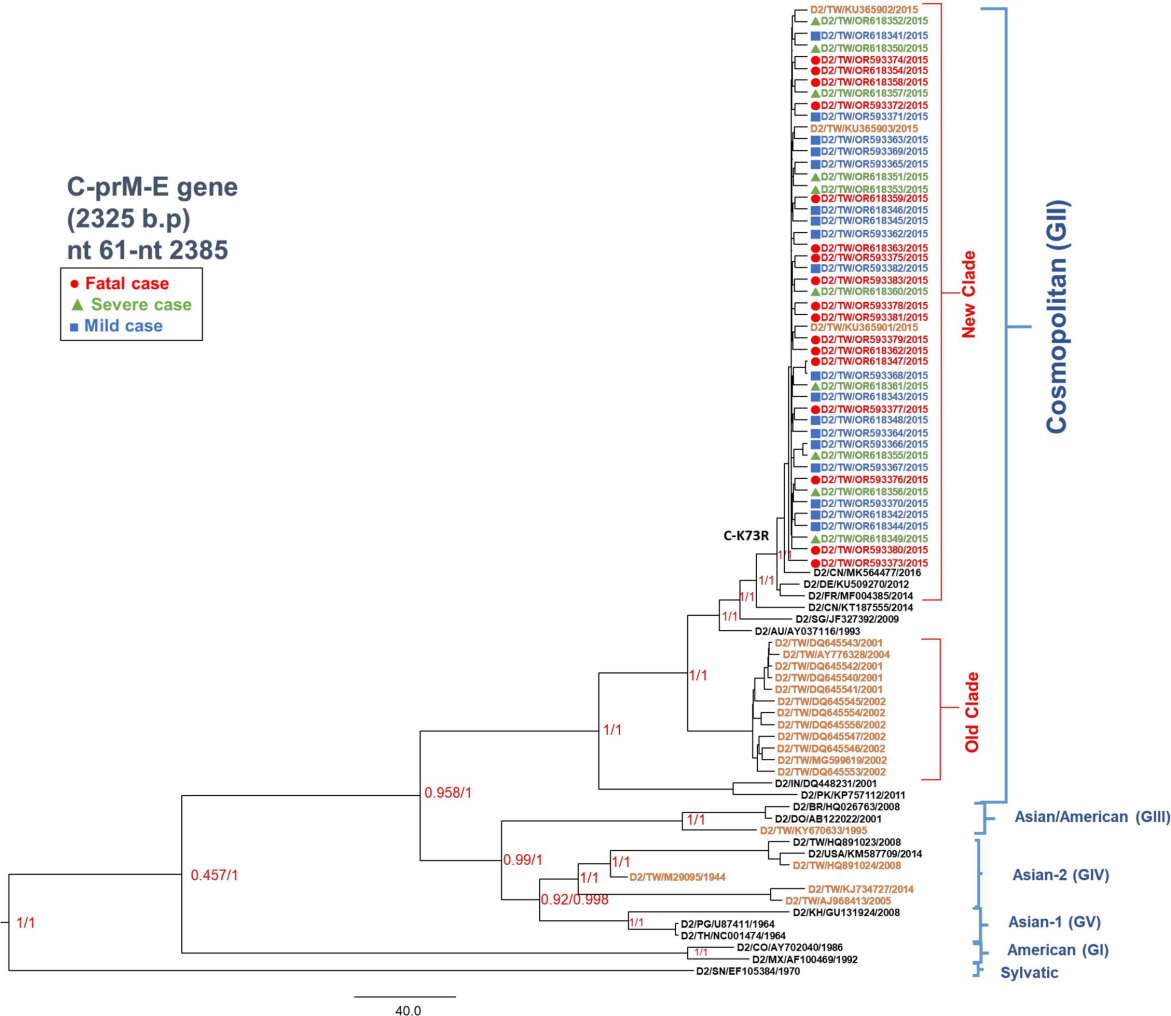
Bayesian evolutionary analysis of DENV-2 isolates based on C to E gene. The model was evaluated using a relaxed uncorrelated lognormal molecular clock (local clock model) and a strict clock (global clock model) with a prior Bayesian skyline coalescent. Forty-five C-prM-E gene sequences (nt 61 to nt 2385) from isolated strains and 38 reference sequences from GenBank were used for phylogenetic tree reconstructions. Six genotypes were assigned according to previous studies. The fatal cases are indicated by red circles, the severe cases are indicated by green triangles, and the mild cases are indicated by blue squares. The reference strains of Taiwan were indicated by the orange font. The D2/EF105384/1970, was used as an out-group. The nodes indicate the node heights. The posterior probabilities for each node were indicated as X/Y, where X indicates the probability for lognormal and Y indicates the probability for the global clock.

Bayesian evolutionary analysis based on the sequences of C-prM-E structural protein genes revealed that all 45 isolates from the 2015 Tainan epidemic were assigned to one cluster that was classified as DENV-2 Cosmopolitan genotype (Fig 1), which was consistent with the phylogenetic analysis of E gene only (S2 Fig). Although the Taiwan DENV-2 isolates from 2001, 2002 and 2004 were also classified as Cosmopolitan genotypes (old clade), the DENV isolates in 2015 were separated into a different cluster (new clade) (Fig 1), suggesting a clade replacement event may have occurred. The results also showed that the most recent origins of the new clade were highly similar to isolates from China in 2014 (D2/CN/KT187555/2014).

### Comparison of amino acid and nucleotide sequence variations

To determine whether there is any significant difference in the C-prM-E gene region of DENV variations between mild, severe, and fatal cases during the 2015 outbreak, the 45 isolates were analyzed in the nucleic acid and amino acid level. The nucleotide and amino acid sequence identity of the C to E gene from 45 isolates ranged from 99.7% to 100% and 99.96% to 100%, respectively. The nucleotide and amino acid sequence identity with other global isolates of Cosmopolitan genotype ranged from 93.7% to 100% and 98.2% to 100%, respectively.

Comparing the 2015 DENV-2 isolates, we found 21 substitutions (Table 1). These substitutions were located at positions 181, 214, 314, 393, 610, 882, 918, 966, 1057, 1354, 1491, 1635, 1814, 1837, 1955, 2095, 2121, 2127, 2136, 2277, and 2355. From these 21 variations, 4, 3, and 14 variations were found in the C, prM and E protein, respectively. Of these 21 variations, there were only 7 non-synonymous substitutions: C-G214C (G40R) whereby glycine (G) was substituted with arginine (R) at position 40, C-A314C (K73R), prM-C610G (Q58E), E-G1354A (V140M), E-A1814G (Q293R), E-A1837G (M301V), and E-T1955C (M340T) (Table 1).

**Table 1:** List of the 21 substitutions in the 2015 DENV isolates.

Position (nt) ^a^	Protein (position)	Amino acid change ^b^
C181T	C_29_	synonymous substitution
G214C	C_40_	G➔R
A314G	C_73_	K➔R
C393T	C_99_	synonymous substitution
C610G	prM_58_	Q➔E
T882C	prM_148_	synonymous substitution
T918C	prM_160_	synonymous substitution
C966T	E_10_	synonymous substitution
T1057C	E_41_	synonymous substitution
G1354A	E_140_	V➔M
C1491T	E_185_	synonymous substitution
G1635A	E_233_	synonymous substitution
A1814G	E_293_	Q➔R
A1837G	E_301_	M➔V
T1955C	E_340_	M➔T
C2095T	E_387_	synonymous substitution
A2121G	E_395_	synonymous substitution
T2127C	E_397_	synonymous substitution
A2136G	E_400_	synonymous substitution
C2277T	E_447_	synonymous substitution
C2355T	E_473_	synonymous substitution

^**a**^ Nucleotide: nt

^**b**^ Amino acid abbreviations. E: Glutamate; G: Glycine; K: Lysine; M: Methionine; Q:Glutamine; R: Arginine; T: Threonine; V: Valine.

Compared with Cosmopolitan genotype D2/AU/AY037116/1993, various substitutions were found in isolates from patients with different severity from the 2015 outbreak. One variation, C-A314C (K73R), was found in the C protein of all 2015 isolates, while C-G214C (G40R) was found in the mild case D2/TW/18925/2015 (Accession No. OR593362). From a fatal case, D2/TW/27688/2015 (Accession No. OR593383), one variation was found in the prM-region (prM-C610G (Q58E)). Five variations were located in the E protein: one variation E-A1814G (Q293R) from a mild case (D2/TW/16458M8/2015), two variations E-A1837G (M301V) and E-T1955C (M340T) from severe cases (D2/TW/24915/2015 and D2/TW/27168/2015), and two variations E-G1354A (V140M) and E-A1357G (I141V) from a fatal case (D2/TW/28536/2015). Compared with other Cosmopolitan genotypes in the past 10 years in Taiwan, a new substitution C-A314G (K73R) was found persistently in all of the 2015 isolates (Table 2).

**Table 2:** Comparison of the amino acid sequence in DENV-2.

		Gene (position) / substitution of amino acid^a^																						
**ID, state, year of isolation**		**C (1–114)**					**prM (1–166)**					**E (1–495)**												
		**9**	**40**	**73**	**104**	**108**	**29**	**58**	**127**	**148**	**161**	**52**	**71**	**140**	**141**	**149**	**162**	**164**	**293**	**301**	**340**	**390**	**480**	**484**
**American genotype**																								
	**D2/MX/AF100469/1992**	**R**	**G**	**K**	**M**	**L**	**D**	**Q**	**I**	**H**	**I**	**Q**	**D**	**V**	**I**	**H**	**V**	**I**	**Q**	**M**	**M**	**D**	**V**	**I**
	**D2/CO/AY702040/1986**					**V**																		
**Asian genotype 1**																								
	**D2/PG/U87411/1964**	**K**				**V**							**E**				**I**					**N**		
	**D2/KH/GU131924/2008**	**K**				**V**					**V**		**E**		**V**		**I**	**V**				**N**		
**Asian genotype 2**																								
	**D2/PG/M29095/1944**					**V**											**I**					**N**		**V**
	**D2/USA/KM587709/2014**					**V**							**E**				**I**					**N**		**V**
**Asian/American genotype**																								
	**D2/DO/AB122022/2001**				**V**	**V**							**E**				**I**				**T**	**N**		**V**
	**D2/BR/HQ026763/2008**				**V**	**V**							**E**				**I**				**T**	**N**		**V**
**Cosmopolitan genotype**																								
	**D2/AU/AY037116/1933**				**I**	**M**	**N**		**V**	**Y**	**V**	**H**	**A**			**N**	**I**	**V**				**S**		**V**
	**D2/IN/DQ448231/2001**				**V**					**Y**	**V**		**A**		**V**	**N**	**I**	**V**				**S**		**V**
	**D2/TW/DQ645540/2001**				**V**	**M**	**N**		**V**	**Y**		**H**	**A**			**N**	**I**	**V**				**S**		**V**
	**D2/TW/DQ645545/2002**				**V**	**M**	**N**		**V**	**Y**		**H**	**A**			**N**	**I**	**V**				**S**		**V**
	**D2/DE/KU509270/2012**	**K**		R	**I**	**M**	**N**		N	**Y**	**V**	**H**	**A**			**N**	**I**	**V**				**S**	G	**V**
	**D2/FR/MF004385/2014**			R	**I**	**M**	**N**		N	**Y**	**V**	**H**	**A**			**N**	**I**	**V**				**S**		**V**
	**D2/CN/KT187555/2014**				**I**	**M**	**N**		**V**	**Y**	**V**	**H**	**A**			**N**	**I**	**V**				**S**		**V**
	**D2/CN/MK564477/2016**			R	**I**	**M**	**N**		**V**	**Y**	**V**	**H**	**A**			**N**	T	**V**				**S**		**V**
**Isolation from this study**																								
	**D2/TW/16155/2015 (x39)** ^**b**^			R	**I**	**M**	**N**		**V**	**Y**	**V**	**H**	**A**			**N**	**I**	**V**				**S**		**V**
	**D2/TW/18925/2015**		R	R	**I**	**M**	**N**		**V**	**Y**	**V**	**H**	**A**			**N**	**I**	**V**				**S**		**V**
	**D2/TW/16458/2015**			R	**I**	**M**	**N**		**V**	**Y**	**V**	**H**	**A**			**N**	**I**	**V**	R			**S**		**V**
	**D2/TW/24915/2015**			R	**I**	**M**	**N**		**V**	**Y**	**V**	**H**	**A**			**N**	**I**	**V**			T	**S**		**V**
	**D2/TW/27168/2015**			R	**I**	**M**	**N**		**V**	**Y**	**V**	**H**	**A**			**N**	**I**	**V**		V		**S**		**V**
	**D2/TW/27688/2015**			R	**I**	**M**	**N**	E	**V**	**Y**	**V**	**H**	**A**			**N**	**I**	**V**				**S**		**V**
	**D2/TW/28536/2015**			R	**I**	**M**	**N**		**V**	**Y**	**V**	**H**	**A**	M	V	**N**	**I**	**V**				**S**		**V**

^a^Amino acid substitutions compared to D2/MX/AF100469/1992. A: Alanine; D: Aspartate; E: Glutamate; G: Glycine; H: Histidine; I: Isoleucine; K: Lysine; L: Leucine; M: Methionine; N: Asparagine; Q: Glutamine; R: Arginine; S: Serine; T: Threonine; V: Valine.

^b^Total of 39 additional isolates that share a similar sequence.

### Wildtype virus had better replication rates, NS1 secretion, and fitness than rgC-A314G (K73R)

**Fig 2:**
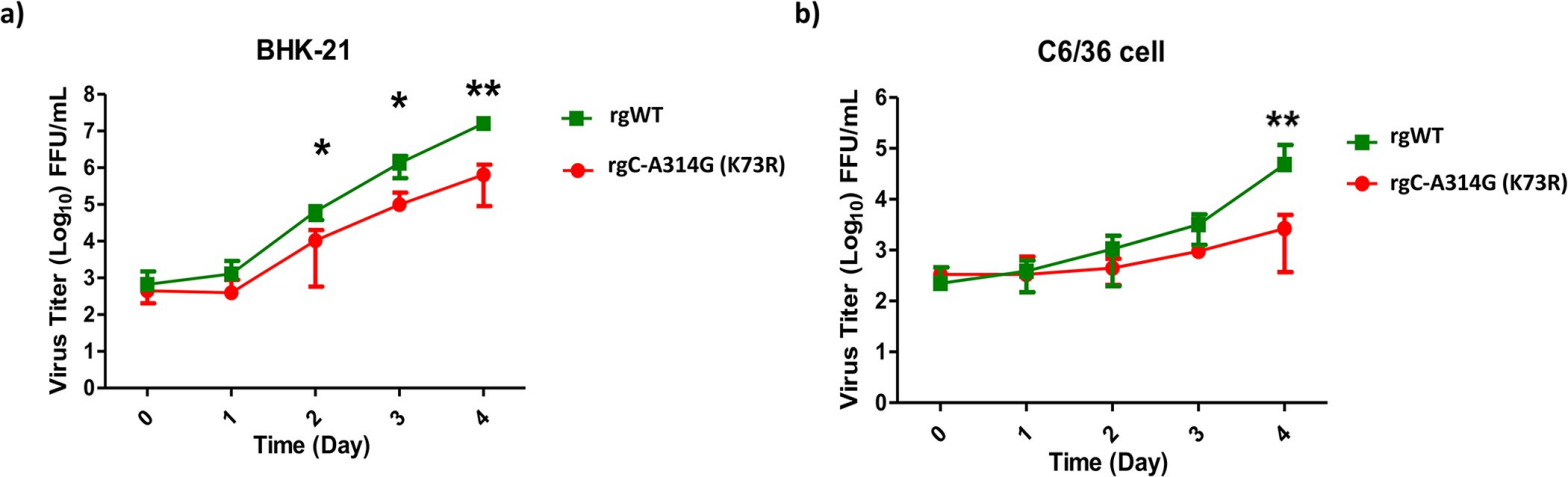
Multiple-step replication cycle of WT and rgC-A314G (K73R). BHK-21 (a) and C6/36 (b) cells were infected with WT virus and rgC-A314G (K73R) at a MOI of 0.01. Virus titers were quantified at 0-, 1-, 2-, 3-, and 4-days post-infection via ELISpot assay. Titers were calculated for FFU/ml. Statistical difference comparing mutant with wildtype was calculated using two-way ANOVA. *p<0.05, **p<0.001.

Based on the analysis of amino acid variations data, the C-A314G (K73R) mutation was persistent in every isolate. We thus hypothesized that the variation C-A314G (K73R) was one of the causes of the DENV-2 outbreak in Tainan in 2015. To investigate the effects of the variation on viral properties, reverse genetics virus was generated by means of site-directed mutagenesis. The multiple-step virus growth kinetics of the virus variant were analyzed and compared with wildtype (WT) virus in various cell lines. BHK-21 and C6/36 cells were infected by viruses with the concentration of low multiplicity of infection (MOI 0.01). The cell lysates were collected daily from 0 to 4 days post-infection (dpi). Viral titers were quantified using ELISpot. In BHK-21 cells, the WT virus had a significantly higher titer than the rgC-A314G (K73R) at 2-, 3-, and 4-days post-infection (Fig 2 and S6 Table). In C6/36 cells, the WT virus had a significantly higher titer than the rgC-A314G (K73R) at 4 dpi. These results indicate that WT virus grew better than rgC-A314G (K73R) in both C6/36 cells and BHK-21 cells.

**Fig 3:**
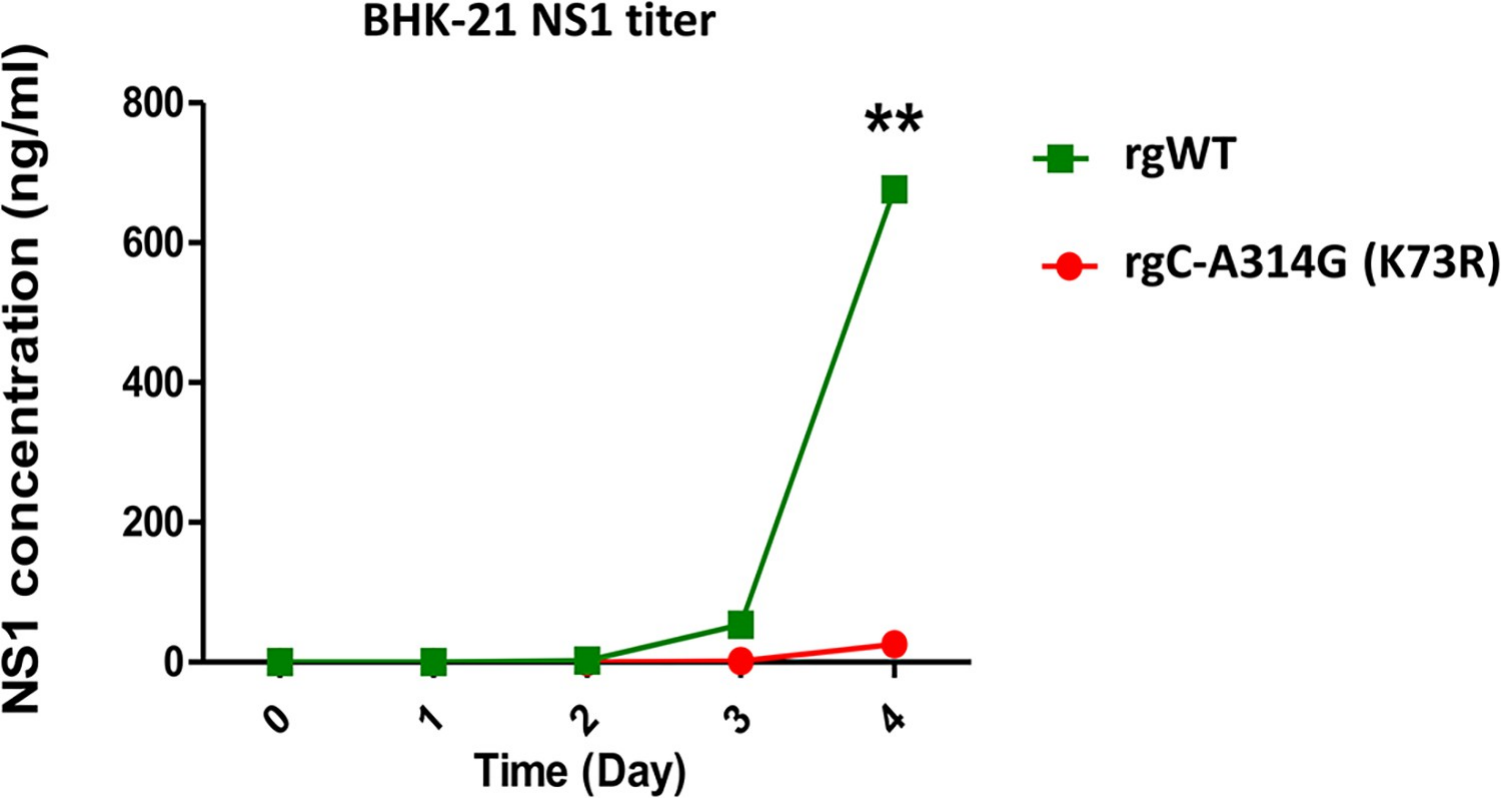
NS1 titer of WT and rgC-A314G (K73R) in BHK-21 cells. BHK-21 cells were infected with viruses containing the WT and rgC-A314G (K73R) at a MOI of 0.01. NS1 concentrations were quantified at 0-, 1-, 2-, 3-, and 4-days post-infection via NS1 Ag ELISA assay. Concentrations were calculated for ng/ml. Statistical difference comparing mutant with wildtype was calculated using two-way ANOVA. **p<0.001.

We further investigated whether the C-A314G (K73R) mutations affect the DENV NS1 protein production. BHK-21 cells were infected with viruses of MOI 0.01 and the cell lysates were quantitated for NS1 by ELISA. In BHK-21 cells, the WT C-314A (K73) virus showed a significantly higher NS1 titer than the virus variant rgC-A314G (K73R) at day 4 post-infection (Fig 3 and S7 Table).

**Fig 4:**
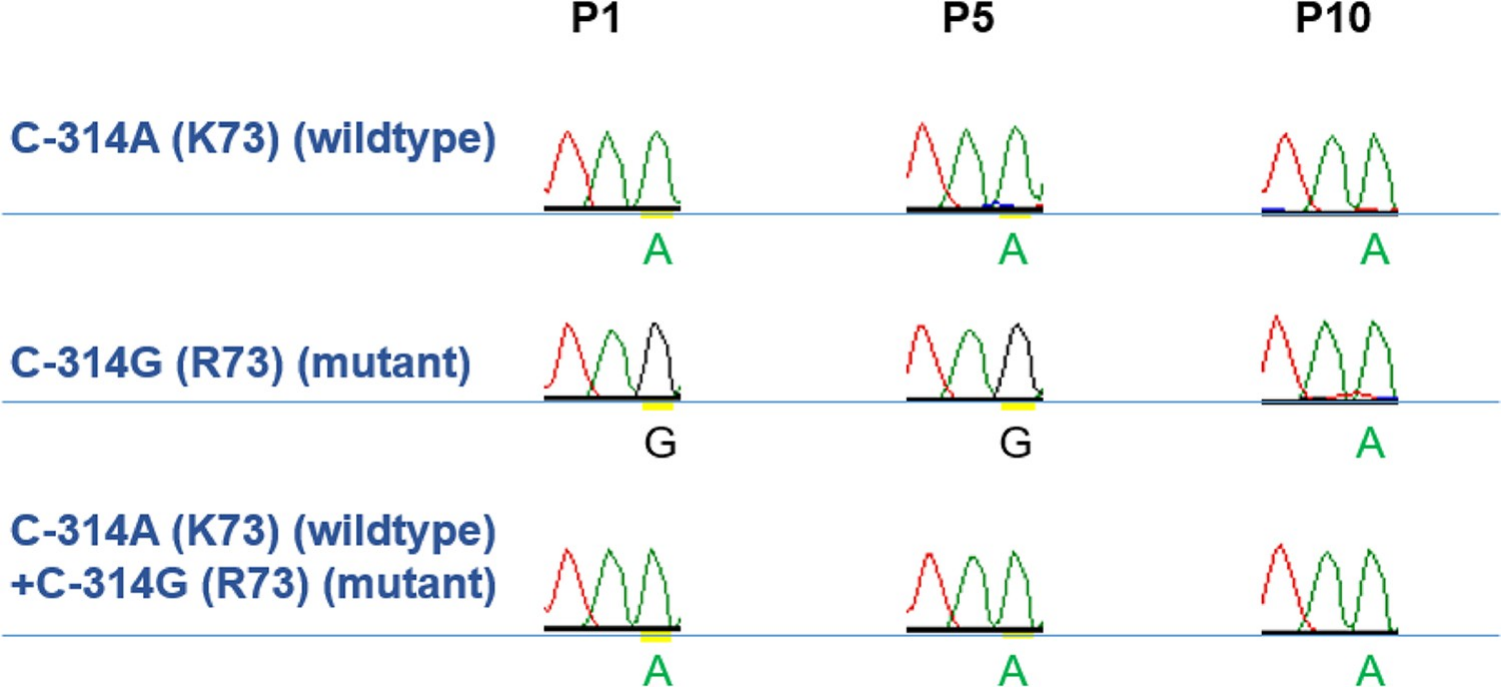
Comparison of virus fitness of variant rgC-A314G (K73R) and WT. Competition assay was utilized to determine the stability of the variant. Mutant virus was mixed with wildtype virus in a ratio of 1:1 and was used to infect BHK-21cells. Viruses were harvested, extracted, and amplified at P1, P5 and P10, and the ratio of the variant was determined using Sanger sequencing (Genomics). The full amino acid codon was not displayed, and the labeled codon represents the second codon of the amino acid.

Competition assay was performed to investigate the fitness of the substitution. BHK-21 cells were infected with a mixture of WT virus and rgC-A314G (K73R) (1:1), WT only, or rgC-A314G (K73R) only. The results showed that the substitution was replaced by wildtype by the first passage (P1) (Fig 4). In the control of rgC-A314G (K73R) only, the substitution remained until the tenth passage (P10) where it eventually reverted to WT (C-314A (K73)). This showed that the substitution was not able to be retained in a competition or mixed population.

### Haplotype analysis of DENV-2 collected at different time-points

DENV, being an RNA virus, tends to have a high mutation rate due to the low fidelity of the RdRp. In the quasispecies theory, genetic diversity caused by the low-fidelity polymerase of DENV is advantageous for the virus population to survive in changing environments. In the population wide analysis, the C-A314G (K73R) mutation was found in every isolate, regardless of the level of disease severity. As infection progresses, the genetic diversity of the viral population can change. Therefore, we further investigated the changes in virus population within a single DENV-2-infected patient to examine the difference in genetic variations. We thus sequenced DENV-2 infected patient isolates by NGS using the Illumina Miseq platform.

**Fig 5:**
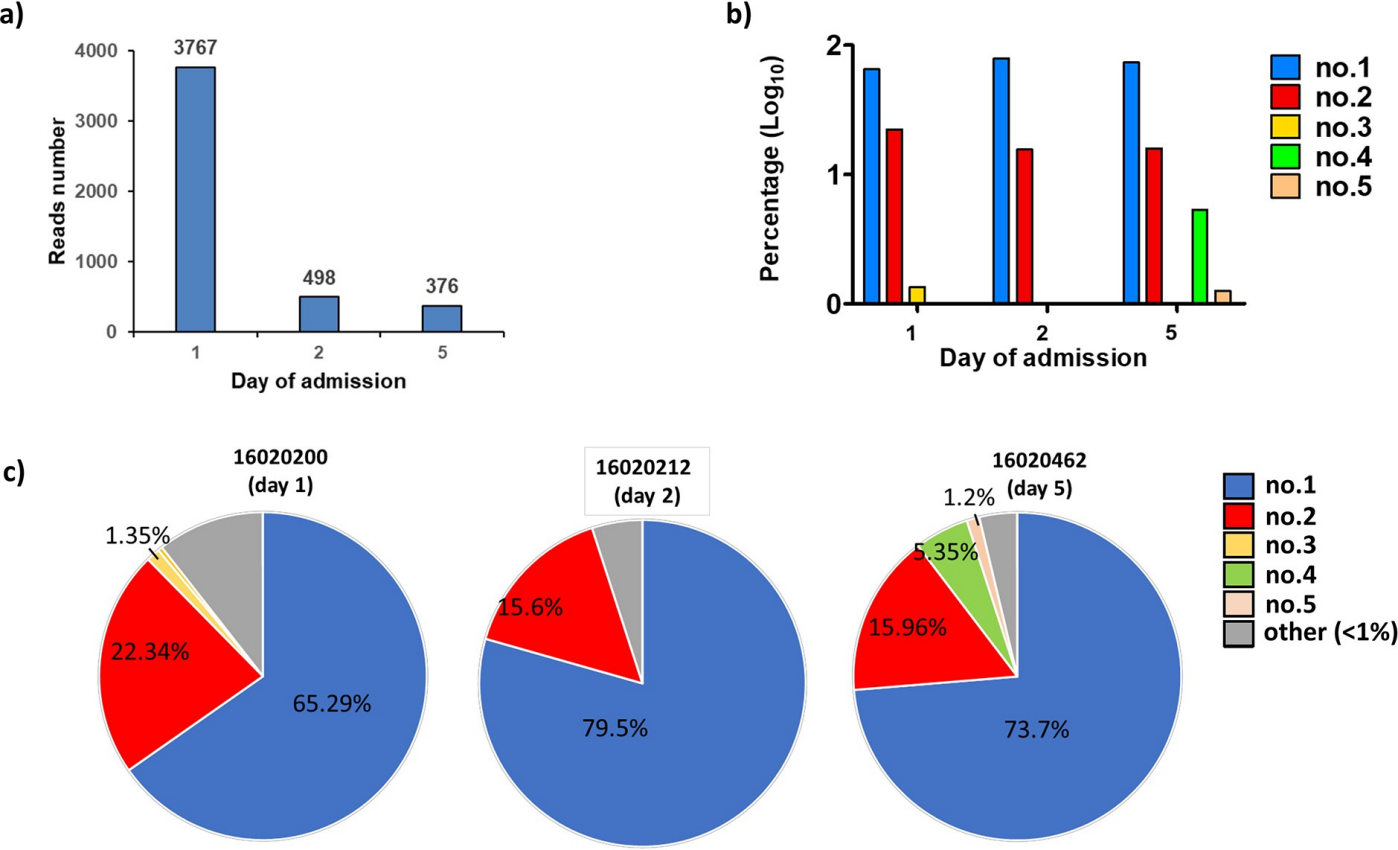
Haplotypes of DENV-2 infected cases. The proportion of haplotypes within one DENV-2 infected patient. (a) The total number of haplotypes on the different days were shown. The percentage of haplotypes were calculated at 1-, 2- and 5-days post-admission via QuasiRecomb. (b) Only the percentage of haplotypes with proportions greater than 1% were included in the haplotype analysis. (c) Pie chart representation of haplotype proportions of DENV-2 samples 16020200, 16020212, and 16020462 collected at 1-, 2- and 5-days post-admission, respectively. Colors of each pie section represent the same haplotype except for the pie section represented by gray (others, <1%).

The virus population in quasispecies is comprised of many variants, also known as haplotypes. To analyze the haplotypes within the viral population, NGS data were refined by QuasiRecomb, duplicate reads were removed and haplotypes with proportions ≥1% were analyzed. Prior to refinement, the resulting number of reads for the DENV-2 samples collected from the same patient at 1-, 2-, and 5-days post-admission were 766144, 812273, and 882418, respectively. After refining the reads to obtain the more unique reads, in the haplotype analysis of three DENV-2 samples, the number of haplotypes were 3767, 498, and 376 at days 1, 2 and 5 of admission, respectively (Fig 5A), and decreased with the time post-infection (Fig 5B). On the first day, there were three haplotypes with greater than 1% (Fig 5B and 5C). However, of note, on the second day haplotype no. 3 disappeared and on the fifth day, two new haplotypes (no. 4 and no. 5) emerged. All haplotypes were of the Cosmopolitan genotype (S3 Fig).

Differences in nucleotide and amino acids between the haplotypes were shown in Table 3. On the first day, compared with haplotype no. 1, both haplotype no. 2 and no. 3 had one synonymous substitution at NS4A-G6366A and NS2B-T4425C, respectively (Table 3). On the fifth day, new haplotypes possessing a new nonsynonymous substitution NS4A –C6694A (L115I) occurred in both haplotypes no. 4 and no. 5. In addition, haplotype no. 5 had a synonymous substitution in the NS4A region (NS4A-G6366A) similar to haplotype no. 2. All haplotypes were classified as DENV-2 Cosmopolitan genotype and were closely related to strains from China in 2014. These results show that as DENV infection progresses, the viral population remains ever changing with the emergence of new haplotypes and variants.

**Table 3:** Intrahost genetic diversity during DENV-2 infection course.

Amino acid position	NS2B-107	NS4A-5	NS4A-115
Nucleotide positon*	4425	6366	6694
Haplotype no. 1^a^	Phe	Glu	Leu
	TTT	GAG	CTC
Haplotype no. 2 ^a^	Phe	Glu	Leu
	TTT	GAA	CTC
Haplotype no. 3 ^b^	Phe	Glu	Leu
	TTC	GAG	CTC
Haplotype no. 4 ^c^	Phe	Glu	Ile
	TTT	GAG	ATC
Haplotype no. 5 ^c^	Phe	Glu	Ile
	TTT	GAA	ATC

Phe: Phenylalanine; Glu: Glutamate; Leu: Leucine; Ile: Isoleucine

*The variation position of nucleotide was underlined, and different nucleotide or amino acid are indicated by bold font.

^**a**^Haplotype found in samples from days 1, 2, and 5 post-admission.

^**b**^Haplotype found in sample from day 1 post-admission only.

^**c**^Haplotype found in sample from day 5 post-admission only.

## Discussion

In 2015, a dengue virus serotype 2 Cosmopolitan genotype epidemic occurred in southern Taiwan, namely Tainan and Kaohsiung. In this study, we analyzed the dengue isolates collected during this epidemic. Phylogenetic analysis based on the sequences of C to E genes revealed that all 45 isolates from the 2015 Tainan epidemic were assigned to one cluster that was classified as DENV serotype 2 Cosmopolitan genotype.

Our phylogenetic analysis and sequence similarity analysis, the diversity of the 45 isolates was very low and were similar to the D2/CN/KT187555/2014 isolate from China in 2014 (Fig 1). Sun *et al* reported that the third largest historical outbreak of dengue occurred from July to December 2014, in Guangdong, China [30]. According to their phylogenetic analyses of DENV, it consisted of DENV-1 genotypes I, IV, and V, DENV-2 Cosmopolitan and Asian I genotypes, and DENV-3 genotype III. Schreiber *et al* revealed that the 2005 DENV-1 outbreak that occurred in Singapore showed close relations between the DENV-1 genotype I isolates and Chinese isolates of 2004, thus suggesting the transfer of DENV between China and Singapore [25]. Similarly, with the close relation between the Taiwan 2015 isolates and the China 2014 isolate, transfer of DENV-2 between these two countries may have occurred. Additionally, as the characteristics of the immune response of the population from where the virus is transmitted from, this would influence the variant composition of the virus populations [51].

Based on the DENV-2 analysis of amino acid variations, while there were apparently uncorrelated mutations among the isolates, a predominant mutation C-A314G (K73R) was found in all isolates in 2015 outbreak in Taiwan (Table 2). Zhang *et al* previously reported that in the mid 1990’s, a major DENV-1 clade replacement event occurred in Thailand [52]. Several amino acid changes that may have played a role in this major DENV-1 clade replacement event were identified. Furthermore, higher transmission potential was seen in isolates that belonged to the new clade, with increased infectious virus titers in the hemocoel of infected *Aedes aegypti* mosquitoes [34]. Singapore also experienced a DENV-2 Cosmopolitan genotype clade replacement in 2007 [26]. Similar to this, we noted that the 2015 DENV-2 isolates formed a new clade while another cluster was seen with isolates from the 2001/2002 outbreak (old clade). We thus hypothesized that the variation C-A314G (K73R) may have caused the clade replacement event and may also be one of the causes of the 2015 DENV-2 outbreak in Taiwan.

To test the fitness and stability of the C-A314G (K73R) mutation, viruses (wildtype, mutant, wildtype + mutant) underwent a series of passages (Fig 4). By the 10^th^ passage (P10), the mutant had reverted to wildtype, indicating that the mutation was not stable. Additionally, during a competition where wildtype and mutant virus were added in a 1:1 ratio, the wildtype virus outcompeted the mutant virus from the first passage (P1), despite being on equal footing during infection. Comparing reverse genetics (rg) C-A314G (K73R) virus to wildtype (WT) virus, rgWT had better replication rates, higher NS1 secretion, and better fitness than rgC-A314G (K73R). These results indicated that rgC-A314G (K73R) virus with its slower replication led to decreased NS1 protein titers. Studies have shown that the NS1 protein is not only essential for RNA replication via interaction and formation of the virus replication complex but is also required for the modulation of infectious particle production by interacting with the structural proteins [53–55]. Mutations within the NS1 protein have been found to affect the production of NS1 and its secretion, enhance viral replication, and increase pro-inflammatory cytokine production [56–58]. Therefore, DENV may have other variations in other regions which may have contributed to the outbreak.

During viral transmission between hosts, much of the virus genetic diversity is lost due to selection pressures. Sim *et al* reported that >90% of single nucleotide variants were lost during the transmission of virus from human to mosquito and within the vector from the abdomen to salivary glands [59]. We thus hypothesized that while the C-A314G (K73R) mutation may have been maintained during viral transmission, but due to its attenuated nature, other mutations may be required to work cooperatively during the DENV outbreak. By substituting different segments of a pre-2015 outbreak strain with the respective segments of a 2015 outbreak strain (TW2015), Lin *et al* identified the prM and E genes as key virulence determinants in the host [60]. The TW2015 virus was found to be highly virulent *in vivo* and had high transmissibility to *Aedes* mosquitoes. However, the specific mutations associated with this within the prM and E genes were not identified. The prM and E genes may thus contain mutations that could potentially aid the C-A314G (K73R) mutation but were otherwise not included during the analysis of the mutation alone.

Another possibility for the persistence of the C-A314G (K73R) mutation is the reduction in genetics of the viral population which reflects on the original members of a population which is mainly seen during the transmission of viruses from one host to another. Di Lello *et al* previously reported that during the transmission of hepatitis C virus from one individual to another, the genetic diversity of the virus was found to be decreased [61]. Another study also showed the decrease in genetic diversity of human-immunodeficiency virus (HIV) in HIV-infected individuals that shared needles during drug use [62]. Other studies have also mentioned the spread of virus and the decrease in genetic diversity [63,64]. Therefore, while the genetic diversity of the DENV viral population may have decreased during viral transmission from mosquito vector to human host, the C-A314G (K73R) mutation along with others possibly found in the non-structural region may have been advantageous for virus transmission.

In the quasispecies theory, genetic diversity caused by the low-fidelity polymerase of DENV can be advantageous for the virus population to survive in changing environments. Different mutations between haplotypes interact cooperatively and contribute to the adaptation and evolution of the population as well as viral fitness. In this study, three sera were collected from one DENV-2 infected patient at different time-points (day 1, 2, and 5 post-admission). In the haplotype analysis, the number of haplotypes decreased with the days of admission, and the results showed that the virus population produced was probably influenced by the host environment, which resulted in the selection of virus. On the fifth day, two new haplotypes emerged with >1% population, whereby leucine mutated to isoleucine in NS4A-L115I. This NS4A-L115I mutation was the only non-synonymous mutation found within the viral infrapopulation analysis. While the C-A314G (K73R) mutation may not be important in the pathogenesis within a single infected individual and may require the cooperation of other mutations, the NS4A-L115I mutation appeared later during the progression of infection, suggesting its potential selective advantage during infection.

NS4A induces autophagy which protects cells against death and enhances virus replication and plays a role in the induction of the membrane alterations that are required for virus replication [65]. In contrast, Zou *et al*. in 2015 reported that mutations in NS4A that affects interactions with NS4B abrogated or severely reduced virus replication, which indicated the importance of NS4A and its interaction with NS4B in dengue reproduction [66]. Amino acid substitution of these NS4A residues exhibited distinct effects on viral replication. Three of the four NS4A mutations (L48A, T54A, and L60A) that affected the NS4A-NS4B interaction abrogated or severely reduced viral replication [66]. Jia *et al* reported that two different variants which were called M2 and M14 displayed a lethal phenotype due to the impairment of RNA replication [67]. However, these two variants could rescue viral RNA replication by an NS4A-A21V mutation. In our study, the results revealed that DENV-2 continually adapts to the host environment through the diversity of quasispecies. As for the NS4A-L115I mutation that occurred on the fifth day, additional research is required to determine whether this variation has any impact on viral replication.

Apart from DENV-2, a single DENV-1 (D1/15016360/TW/2015) was identified in 2015 at NCKUH, Tainan. The DENV-1 isolate had a total of 4410 haplotypes, with only 6 having proportions ≥1% (S4A Fig and S7 Table), with all being classified as DENV-1 genotype I and were highly similar to the D1/TH/JN415527/2008 isolate from Thailand in 2008 (S5 Fig). Two DENV-3 were also isolated at NCKUH in 2016 (D3/16031760/TW/2016) and 2017 (D3/1700643/TW/2017). Each isolate had 2 haplotypes with proportions ≥1% (S4B Fig and S9 Table) that were classified as DENV-3 genotype I and were highly similar to the D3/PH/KU509279/2008 isolate in Philippines and the D3/TW/DQ675519/1955 isolate in Taiwan in 1955 (S6 Fig).

The D1/15016360/TW/2015 has 6 major haplotypes, consisting of 2 synonymous substitutions and 3 nonsynonymous substitutions all in the NS3 region: NS3–G4665C (R49T), NS3–G4665A (R49K) and C5073A (P175H) (S8 Table). This indicated that D1/15016360/TW/2015 was possibly able to adapt to the pressures in the host environment through mutations of the NS3 region. Contrastingly, D3/16031760/TW/2016 and D3/1700643/TW/2017 only showed a single synonymous mutation amongst the haplotypes in the NS4A region (S9 Table).

Ali *et al*. in 2015 reported that all DENV-2 isolates were divided into seven main groups containing five previously defined genotypes [68]. Comparison of the amino acid sequences showed specific mutations among the various groups of DENV-2 isolates. The majority of amino acid mutations were observed in the NS5 gene, followed by the NS2A, NS3, and NS1 genes, while the smallest number of amino acid substitutions was recorded in the capsid gene, followed by the prM/M, NS4A, and NS4B genes. Maximum evolutionary distances were found in the NS2A gene, followed by the NS4A and NS4B genes. Correspondingly, in our analysis of DENV, the variations were all located in the nonstructural region of the genome, namely NS2B, NS3, and NS4A which are important for various enzymatic functions needed during the viral life cycle. These reports suggested that DENV is probably able to defend against or adapt to the host environment through the nonstructural gene mutations.

It is worth noting that during the process of virus amplification, cell passages may also result in the accumulation of adaptive mutations that are not naturally found. Additionally, further amplification via PCR can also result in the production of mutations. Viral passages were kept at a minimum (2 passages) to reduce the risk of adaptive mutations occurring. Therefore, the conditions during cell passage virus amplification and PCR were the same for each isolate, thus keeping the standard across the study. Furthermore, the 45 isolates were only sequenced from C to E gene. It is unknown whether there are other persistent mutations within the nonstructural region of the 2015 isolates that may have either played a role in the outbreak and/or disease severity, or that work in conjunction with the C-A314G (K73R) mutation.

In conclusion, the phylogenetic analysis, a total of 45 DENV-2 outbreak isolates were assigned to one cluster belonging to the Cosmopolitan genotype. The new substitution C-A314G (K73R) in the capsid region was found from isolates in the 2015 outbreak, however, the replication rate and NS1 titer of the variant C-A314G (K73R) were reduced when compared with wildtype, thus suggesting that there may be other variations in other regions that contributed to the 2015 DENV outbreak in Taiwan. While the C-A314G (K73R) was found in all 45 isolates, analysis of isolates collected from a single patient at different time-points showed the emergence of new haplotypes within the viral population. In our NGS data analysis of the DENV-2 isolates from the same patient, the variations were all located in the nonstructural region of the genome, with one mutation (NS4A-L115I) emerging in haplotypes found on day 5 post-admission. Therefore, while C-A314G (K73R) may have appeared only in isolates from 2015 and not prior, the mutation alone would not be enough to further the outcome of disease severity but may require the aid of other mutations such as NS4A-L115I within the viral population. All results demonstrated that DENV continually adapts between inter-and intra-host.

## Supporting information

S1 Table List of sequencing C-prM-E region of 45 DENV-2 strains. (DOCX)

S2 Table List of samples categorized as secondary infections. (DOCX)

S3 Table RT-PCR and DNA sequencing of C-prM-E gene of DENV-2. (DOCX)

S4 Table DENV Full Genome Amplification. (DOCX)

S5 Table DENV Site-Directed Mutagenesis Primers. (DOCX)

S6 Table Viral titers of wildtype and mutant viruses during growth replication. (DOCX)

S7 Table NS1 titers of wildtype and mutant viruses in BHK-21 cells. (DOCX)

S8 Table Sequence variations in amino acid and nucleotides of the coding region identified in quasispecies of DENV-1. (DOCX)

S9 Table Sequence variations in amino acid and nucleotides of the coding region identified in quasispecies of DENV-3 in two different cases. (DOCX)

S1 Fig Viral load analysis of mild, severe, and fatal case isolates. Viral loads of mild, severe, and fatal cases (a) The number of specimens classified as mild (green), severe (blue), or fatal (red) were grouped into low and high viral load groups. (b) The percentage of the overall number of specimens within the low and high viral groups. (c) The viral load of each specimen was plotted and divided between low (closed symbols) and high (open symbols) viral loads. Student’s t-test was used to analyze the difference between two groups. **p<0.01, ***p<0.001.
(TIFF)

S2 Fig Bayesian evolutionary analysis of 2015 DENV-2 isolates based on E gene. The model was evaluated using a relaxed uncorrelated lognormal molecular clock with a prior Bayesian skyline coalescent. Forty-five E gene sequences (nt 61 to nt 2385) from isolated strains and 38 reference sequences from GenBank were used for phylogenetic tree reconstructions. Six genotypes were assigned according to previous studies. The fatal cases are indicated by red circles, the severe cases are indicated by green triangles, and the mild cases are indicated by blue squares. Mild, severe, and fatal case isolates were assigned to the Cosmopolitan genotype of DENV-2. The reference strains of Taiwan were indicated by the orange font. The D2/EF105384/1970, was used as an out-group.
(TIFF)

S3 Fig Bayesian evolutionary analysis of DENV-2 isolated from one patient in 2016 in Tainan, Taiwan. E gene sequences of 9 haplotypes from 3 samples obtained from a single DENV-2 infected patient at different time-points and 41 reference sequences from GenBank were used for phylogenetic tree reconstructions. Six genotypes were assigned according to previous studies. The patient case in 2016 at days 1, 2 and 5 of admission were labeled by red circles, purple triangles, and blue squares, respectively, and were assigned to the Cosmopolitan genotype of DENV-2. The 2015 isolates in Taiwan are indicated by green font, and the 1995–2014 strains in Taiwan were indicated by the orange font. D2/SNEF105384/1970 was used as an out-group.
(TIFF)

S4 Fig Haplotypes of DENV-1 and DENV-3 Cases. Proportion of haplotypes within each isolate in (a) DENV-1 and (b) DENV-3 cases. Each pie chart represents a single isolate. The different sections within the chart each represent a percentage of a haplotype, whereby the bigger the section the higher the percentage. The major haplotype is represented by the section with the highest percentage. Similar section color does not represent the same haplotype. Each section represents a single individual haplotype.
(TIFF)

S5 Fig Bayesian evolutionary analysis of DENV-1 isolated from a patient in 2015 in Tainan, Taiwan. E gene sequences of 6 haplotypes from one DENV-1 infected patient and 70 reference sequences from GenBank were used for phylogenetic tree reconstructions. Six genotypes were assigned according to previous studies and are highlighted in different colors. The 2015 DENV-1 infected patient case haplotypes were labeled by red circles and red text. The strain D1/BN/KR919820/2014 was used as an out-group.
(TIFF)

S6 Fig Bayesian evolutionary analysis of DENV-3 isolated in 2016–2017 in Tainan, Taiwan. E gene sequences of 4 haplotypes from two DENV-3 infected patients in 2016–2017 and 99 reference sequences from GenBank were used for phylogenetic tree reconstructions. Five genotypes were assigned according to previous studies and are highlighted in different colors. The 2016 DENV-3 case haplotypes were labeled with red circles and red text, and the 2017 DENV-3 case haplotypes were labeled by green squares and green text. The strains D3/PR/AY146762/1963 and D3/PR/AY146761/1977 were used as out-groups.
(TIFF)
