# Supplementary material for: The molecular epidemiology of a dengue virus outbreak in Taiwan: population wide versus infrapopulation mutation analysis
Source: PLoS Negl Trop Dis. 2024 Jun 13;18(6):e0012268. doi: 10.1371/journal.pntd.0012268 (PMC11207123; doi:10.1371/journal.pntd.0012268)
Supplement: S1 Table — (DOCX) [file pntd.0012268.s001.docx]

S1 Table. List of Sequencing C-prM-E region of 45 DENV-2 strains

| Group | No. of specimens in various viral load levels (copies/mL) | | Total |
| --- | --- | --- | --- |
|  | 10^3^-10^6^ | 10^6^-10^9^ |  |
| Mild case | 14 | 4 | 18 |
| Severe case | 4 | 6 | 10 |
| Fatal case | 7 | 10 | 17 |
| Total |  |  | 45 |
