# Supplementary material for: The molecular epidemiology of a dengue virus outbreak in Taiwan: population wide versus infrapopulation mutation analysis
Source: PLoS Negl Trop Dis. 2024 Jun 13;18(6):e0012268. doi: 10.1371/journal.pntd.0012268 (PMC11207123; doi:10.1371/journal.pntd.0012268)
Supplement: S2 Table — (DOCX) [file pntd.0012268.s002.docx]

S2 Table. List of samples categorized as secondary infections

| **Sample** | **Disease severity** | **Rapid IgM^a^** | **Rapid IgG** | **SD ELISA IgM^b^** | **SD ELISA IgG** | **SD IgM/IgG^c^** |
| --- | --- | --- | --- | --- | --- | --- |
| **D2/TW/25793/2015** | Fatal | Pos^d^ | Pos | Neg^e^ | Neg | 5.274 |
| **D2/TW/19823/2015** | Fatal | Neg | Pos | N/A^f^ | N/A | N/A |
| **D2/TW/21719/2015** | Fatal | Neg | Pos | N/A | N/A | N/A |

^a^Samples tested positive for rapid IgM only were classified as primary infections, and rapid IgG only were classified as secondary infections.

^b^Standard Diagnostics Inc (SD) ELISA IgM and IgG are tested only when rapid IgM and IgG are positive.

^c^IgM/IgG ratio of ≥1.14 is used as a cut-off point to determine DENV secondary infection.

^d^Test result is positive (pos).

^e^Test result is negative (neg).

^f^Test result is not available (N/A).
