# Supplementary material for: The molecular epidemiology of a dengue virus outbreak in Taiwan: population wide versus infrapopulation mutation analysis
Source: PLoS Negl Trop Dis. 2024 Jun 13;18(6):e0012268. doi: 10.1371/journal.pntd.0012268 (PMC11207123; doi:10.1371/journal.pntd.0012268)
Supplement: S3 Table — (DOCX) [file pntd.0012268.s003.docx]

S3 Table. RT-PCR and DNA sequencing of C-prM-E gene of DENV-2

| **Primer Name** | **Sequence (5' to 3')** |
| --- | --- |
| D2-14F | ACG TGG ACC GAC AAA GAC AGA TTC |
| D2-1157F | GCC CAA CAC AAG GRG AAC CCA |
| D2-1232R | TGT CTA CCA TGG AGT GTT TGC AG |
| D2-1572R | CAT TGC CTG TGC ACC AGC CAA GC |
| D2-2162R | GCT CCY CTC ATT GTT GTC TC |
| D2-2610R | CAR TCT TGT TAC TGA GCG GA |
