# Supplementary material for: The molecular epidemiology of a dengue virus outbreak in Taiwan: population wide versus infrapopulation mutation analysis
Source: PLoS Negl Trop Dis. 2024 Jun 13;18(6):e0012268. doi: 10.1371/journal.pntd.0012268 (PMC11207123; doi:10.1371/journal.pntd.0012268)
Supplement: S4 Table — (DOCX) [file pntd.0012268.s004.docx]

S4 Table. DENV Full Genome Amplification

| **Serotype** | **Primer Name** | **Sequence (5' to 3')** |
| --- | --- | --- |
| DENV-1 | D1Fw1 | AGT TGT TAG TCT ACG TGG ACC G |
|  | D1Rv3 | ACT CCA CCA GTC AAC ACA GCT AT |
|  | D1Fw4 | TGC ACA ACT CCG AAC AAG GAG G |
|  | D1Rv5 | TGG CGT TCT GTG CCT GGA ATG AT |
| DENV-2 | D2Fw1 | AGT TGT TAG TCT ACG TGG ACC G |
|  | D2Rv3 | CCA GTA TTA TTG AAG CTG CTA TCC A |
|  | D2Fw4 | AAT CAC AGA AAT GGG TAG GCT |
|  | D2Rv5 | TGG CGT TCT GTG CCT GGA ATG AT |
| DENV-3 | D3Fw1 | TAC GTG GAC CGA CAA GAA CAG TTT |
|  | D3Rv3 | ACT ATA GCC GAC GCG ATC CAT TG |
|  | D3Fw4 | CAT GCA GTG GAG GAA CTA CCA GA |
|  | D3Rv5 | TGG CGT TCT GTG CCT GGA ATG AT |
