# Supplementary material for: The molecular epidemiology of a dengue virus outbreak in Taiwan: population wide versus infrapopulation mutation analysis
Source: PLoS Negl Trop Dis. 2024 Jun 13;18(6):e0012268. doi: 10.1371/journal.pntd.0012268 (PMC11207123; doi:10.1371/journal.pntd.0012268)
Supplement: S5 Table — (DOCX) [file pntd.0012268.s005.docx]

S5 Table. DENV Site-Directed Mutagenesis Primers

| **Primer Name** | **Sequence (5' to 3')** |
| --- | --- |
| SING-D2-1-F | GAC GAA TTC TCT AGA TAT CGC TCA ATA |
| SING-D2-593-R | CAG GAA TCG AAT GCA ACC GGC |
| SING-D2-501-F | CAG CAT TCC AGG TAT TAG AAG AAT ATC C |
| SING-D2-971-F | TGC AGT TTC ACT TGA TGC TCG ATG AG |
| SING-D2-1479-F | CGG TGT AGG TCG TTC GCT CCA A |
| SING-D2-1904-F | TGA TTG CAG TCC AGT TAC GCT GGA GTC |
| SING-D2-Sac-I-F | GCC TAT ATA AGC AGA GCT CGT TTA GTG AAC CGT |
| SING-D2-3506-F | GCC AAG AAC ACC CCC TTT AAC AT |
| SING-D2-3648-F | GTT TCC TAA CAA TCC CAC CAA CAG C |
| SING-D2-3702-R | ACA TTA ATA GCT TTT GAT TTT CTA ATT GTT CCC |
| SING-D2-3702-F | GAG ATG GGG AAC AAT TAG AAA ATC AAA AGC TAT |
| SING-D2-3757-R | ATC CTT CCA ATC TCT TTC CTG AAC C |
| SING-D2-4030-R | GCA CCA ACA GTC TAT GTC TTC |
| SING-D2-ScaI-R | GCT TTG CCT CTA TAC AGT ACT TCC TTA GGG TGG |
| SING-D2-4411-F | CTG TGT GAC GAC GAT GGC AAA |
| SING-D2-4793-R | TCC TGT GTC ATT TCC GAC TGC ATG |
| SING-D2-3830-R | GGA ACG CCA TCA CTG TTG GA |
|  |  |
