# Supplementary material for: The molecular epidemiology of a dengue virus outbreak in Taiwan: population wide versus infrapopulation mutation analysis
Source: PLoS Negl Trop Dis. 2024 Jun 13;18(6):e0012268. doi: 10.1371/journal.pntd.0012268 (PMC11207123; doi:10.1371/journal.pntd.0012268)
Supplement: S6 Table — (DOCX) [file pntd.0012268.s006.docx]

S6 Table. Viral titers of wildtype and mutant viruses during growth replication

| **Cell** | **dpi^a^** | **Virus titer (FFU/mL)^b^** | | | | | | |
| --- | --- | --- | --- | --- | --- | --- | --- | --- |
|  |  | **rgWT** | | |  | **rgC-A314G (K73R)** | | |
| **BHK-21** | **0** | 333 | 1×10^3^ | 667 |  | 500 | 500 | 333 |
|  | **1** | 1.67×10^3^ | 1.67×10^3^ | 527 |  | 500 | 527 | 167 |
|  | **2** | 7.38×10^4^ | 5.27×10^4^ | 6.85×10^4^ |  | 5.8×10^3^ | 1.26×10^4^ | 1.26×10^4^ |
|  | **3** | 9.49×10^5^ | 1.48×10^6^ | 1.53×10^6^ |  | 8.96×10^4^ | 5.8×10^4^ | 1.48×10^5^ |
|  | **4** | 1.53×10^7^ | 1.79×10^7^ | 1.48×10^7^ |  | 4.17×10^5^ | 8.67×10^5^ | 6.67×10^5^ |
| **C6/36** | **0** | 167 | 167 | 333 |  | 333 | 333 | 333 |
|  | **1** | 500 | 333 | 333 |  | 333 | 167 | 500 |
|  | **2** | 1.17×10^3^ | 667 | 1.33×10^3^ |  | 500 | 333 | 500 |
|  | **3** | 3.33×10^3^ | 3.83×10^3^ | 2.33×10^3^ |  | 833 | 1×10^3^ | 1×10^3^ |
|  | **4** | 2.67×10^4^ | 7.91×10^4^ | 3.83×10^4^ |  | 3.69×10^3^ | 2.11×10^3^ | 2.11×10^3^ |

^a^ dpi: days post-infection

^b^ Virus titers were quantified via ELISpot in duplicates.
