# Supplementary material for: The molecular epidemiology of a dengue virus outbreak in Taiwan: population wide versus infrapopulation mutation analysis
Source: PLoS Negl Trop Dis. 2024 Jun 13;18(6):e0012268. doi: 10.1371/journal.pntd.0012268 (PMC11207123; doi:10.1371/journal.pntd.0012268)
Supplement: S7 Table — (DOCX) [file pntd.0012268.s007.docx]

S7 Table. NS1 titers of wildtype and mutant viruses in BHK-21 cells

| **dpi^a^** | **NS1 titer (ng/ml)** | | | |
| --- | --- | --- | --- | --- |
|  | **rgWT** | | **rgC-A314G (K73R)** | |
| **0** | 0 | 0 | 0 | 0 |
| **1** | 0 | 0 | 0 | 0 |
| **2** | 2.22 | 2.22 | 0 | 0 |
| **3** | 53.5 | 53.5 | 1.54 | 1.54 |
| **4** | 676.17 | 676.16 | 25.75 | 25.75 |

^a^ dpi: days post-infection
