# Supplementary material for: The molecular epidemiology of a dengue virus outbreak in Taiwan: population wide versus infrapopulation mutation analysis
Source: PLoS Negl Trop Dis. 2024 Jun 13;18(6):e0012268. doi: 10.1371/journal.pntd.0012268 (PMC11207123; doi:10.1371/journal.pntd.0012268)
Supplement: S8 Table — (DOCX) [file pntd.0012268.s008.docx]

S8 Table. Sequence variations in amino acid and nucleotides of the coding region identified in quasispecies of DENV-1

| Amino acid position | NS3-49 | NS3-52 | NS3-175 | NS3-418 |
| --- | --- | --- | --- | --- |
| Nucleotide position* | 4665 | 4675 | 5073 | 5773 |
| Haplotype no. 1 | Arg | Val | Pro | Phe |
|  | AGG | GTC | CCC | TTC |
| Haplotype no. 2 | Thr | Val | Pro | Phe |
|  | ACG | GTC | CCC | TTC |
| Haplotype no. 3 | Arg | Val | Pro | Phe |
|  | AGG | GTA | CCC | TTC |
| Haplotype no. 4 | Arg | Val | Pro | Phe |
|  | AGG | GTC | CCC | TTT |
| Haplotype no. 5 | Arg | Val | His | Phe |
|  | AGG | GTC | CAC | TTC |
| Haplotype no. 6 | Lys | Val | Pro | Phe |
|  | AAG | GTC | CCC | TTC |

Arg: Arginine; Val: Valine; Pro: Proline; Phe: Phenylalanine; His: Histidine; Thr: Threonine.

^*^The variation position of nucleotides was underlined, and different nucleotides or amino acids are indicated by bold font.
