# Supplementary material for: The molecular epidemiology of a dengue virus outbreak in Taiwan: population wide versus infrapopulation mutation analysis
Source: PLoS Negl Trop Dis. 2024 Jun 13;18(6):e0012268. doi: 10.1371/journal.pntd.0012268 (PMC11207123; doi:10.1371/journal.pntd.0012268)
Supplement: S9 Table — (DOCX) [file pntd.0012268.s009.docx]

S9 Table. Sequence variations in amino acid and nucleotides of the coding region identified in quasispecies of DENV-3 in two different cases

| Sample ID  / haplotype no. | Amino acid position | NS4A-108 |
| --- | --- | --- |
|  | Nucleotide position* | 6673 |
| 1700643 | Haplotype no. 1 | Ala |
|  |  | GCC |
|  | Haplotype no. 2 | Ala |
|  |  | GCT |
| 16031760 | Haplotype no. 1 | Ala |
|  |  | GCC |
|  | Haplotype no. 2 | Ala |
|  |  | GCT |

Ala: Alanine

^*^The variation position of nucleotides was underlined, and different nucleotides are indicated by bold font.
