# Supplementary figures and images for: The molecular epidemiology of a dengue virus outbreak in Taiwan: population wide versus infrapopulation mutation analysis
Source: PLoS Negl Trop Dis. 2024 Jun 13;18(6):e0012268. doi: 10.1371/journal.pntd.0012268 (PMC11207123; doi:10.1371/journal.pntd.0012268)

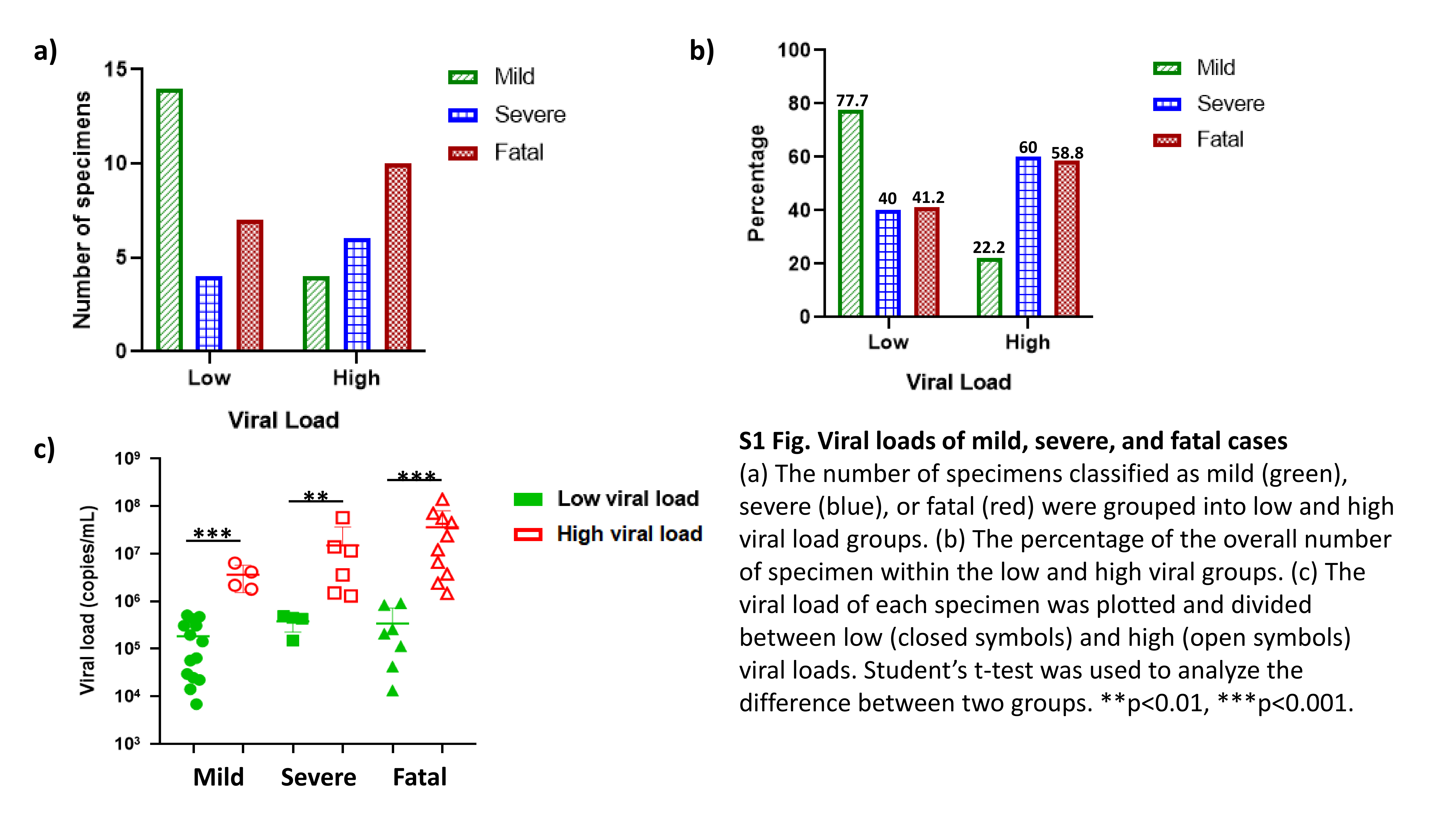

Supplement: S1 Fig — Viral loads of mild, severe, and fatal cases (a) The number of specimens classified as mild (green), severe (blue), or fatal (red) were grouped into low and high viral load groups. (b) The percentage of the overall number of specimens within the low and high viral groups. (c) The viral load of each specimen was plotted and divided between low (closed symbols) and high (open symbols) viral loads. Student’s t-test was used to analyze the difference between two groups. **p<0.01, ***p<0.001. (TIFF) [file pntd.0012268.s010.tiff]
